# Factors associated with the incidence of pressure wounds in critical patients: a cohort study

**DOI:** 10.1590/0034-7167-2021-0267

**Published:** 2022-06-24

**Authors:** Andreza de Oliveira Teixeira, Lídia Miranda Brinati, Luana Vieira Toledo, José Faustino da Silva, Daniela Lucas de Paula Teixeira, Carla de Fátima Januário, Letícia Marques da Silva, Patrícia de Oliveira Salgado

**Affiliations:** I Unidade Básica de Saúde de Canaã. Canaã, Minas Gerais, Brazil.; II Centro Universitário UNIFAMINAS. Muriaé, Minas Gerais, Brazil.; III Universidade Federal de Viçosa. Viçosa, Minas Gerais, Brazil.; IV Casa de Caridade de Viçosa, Hospital São Sebastião. Viçosa, Minas Gerais, Brazil.; V Universidade de São Paulo. São Paulo, São Paulo, Brazil.

**Keywords:** Nursing, Pressure Ulcer, Risk Factors, Intensive Care Units, Critical Care, Enfermería, Úlcera por Presión, Factores de Riesgo, Unidades de Cuidados Intensivos, Cuidados Críticos, Enfermagem, Lesão por Pressão, Fatores de Risco, Unidades de Terapia Intensiva, Cuidados Críticos

## Abstract

**Objectives:**

to identify the incidence of pressure wound in critical patients and its associated factors.

**Methods:**

retrospective cohort study, based on the analysis of 369 critical patients’ records. Descriptive and inferential statistics were used, as well as logistic regression.

**Results:**

the incidence of pressure wounds was 11.4%. Patients who had been hospitalized for four days or more (OR 2.99; CI95% 1.15-7.78), used nasoenteric tubes (OR: 3.81; CI95%: 1.4010.38), vesical drainage catheters (OR: 4.78; CI95%: 1.31-17.38) and tracheostomy (OR: 3.64; CI95%: 1.48-8.97) had a higher chance of developing pressure wounds. The mean score of the Braden scale among participants who developed (14.2 points) pressure wounds was statistically different (p<0.001) than that of those who did not (12.3 points).

**Conclusions:**

the incidence of pressure wounds was associated with a higher time in the unit, the use of nasoenteric tubes, vesical drainage catheters, and tracheostomies were associated with a higher time of hospitalization in the unit.

## RESUMO

**Objetivos:**

identificar a incidência de lesão por pressão em pacientes críticos e os fatores associados à sua ocorrência.

**Métodos:**

estudo de coorte retrospectiva, baseando-se na análise dos prontuários de 369 pacientes críticos. Utilizou-se estatística descritiva e inferencial, com regressão logística.

**Resultados:**

a incidência de lesão por pressão foi de 11,4%. Pacientes com internação por mais de quatro dias (OR 2,99; IC95% 1,15-7,78), em uso de cateter nasoentérico (OR: 3,81; IC95%: 1,4010,38), cateter vesical de demora (OR: 4,78; IC95%: 1,31-17,38) e traqueostomia (OR: 3,64; IC95%: 1,48-8,97) apresentaram maior chance de desenvolver lesão por pressão. A pontuação média da escala de Braden entre os pacientes que desenvolveram (14,2 pontos) ou não (12,3 pontos) lesão por pressão foi estatisticamente diferente (p<0,001).

**Conclusões:**

a incidência de lesão por pressão esteve associada ao maior tempo de permanência na unidade, utilização de cateter nasoentérico, cateter vesical de demora e traqueostomia.

## RESUMEN

**Objetivos:**

identificar la incidencia de úlcera por presión en pacientes críticos y los factores relacionados a su ocurrencia.

**Métodos:**

estudio de cohorte retrospectivo, basándose en el análisis de los prontuarios de 369 pacientes críticos. Se utilizó estadística descriptiva e inferencial, con regresión logística.

**Resultados:**

la incidencia de úlcera por presión fue de 11,4%. Pacientes con internación por más de cuatro días (OR 2,99; IC95% 1,15-7,78), en uso de catéter nasoenteral (OR: 3,81; IC95%: 1,40-10,38), catéter vesical de demora (OR: 4,78; IC95%: 1,31-17,38) y traqueostomía (OR: 3,64; IC95%: 1,48-8,97) presentaron mayor chance de desenvolver úlcera por presión. La puntuación mediana de la escala de Braden entre los pacientes que desarrollaron (14,2 puntos) o no (12,3 puntos) úlcera por presión fue estadísticamente diferente (p<0,001).

**Conclusiones:**

la incidencia de úlcera por presión estuvo relacionada al mayor tiempo de permanencia en la unidad, utilización de catéter nasoenteral, catéter vesical de demora y traqueostomía.

## INTRODUCTION

Intensive care units (ICUs) are environments to care for critical patients, whose high risk of death means they need constant interdisciplinary care. As a result, these units are costly, in a unique physical environment that includes high-complexity equipment from a well-trained multidisciplinary team^(1)^.

In these units, many different treatments are carried out to reestablish the vital function of the patients. However, the care provided to the patients make them more vulnerable, due to changes their level of consciousness and to the use of ventilatory support, sedatives, vasoactive drugs, enteral or parenteral nutrition, hemodynamic instability, invasive procedures, long-term movement restrictions, and others^(1,2,3)^.

These critical patients are susceptible to several adverse events, among which the appearance of pressure wounds (PW) stand out. These wounds are defined as the damage in the skin and/or in its underlying soft tissues, usually on top of bone prominence or related to the use of a medical device or other artifact. The lesion can be an erythema on preserved skin or as an open ulcer and can be painful^(4)^. This is an important public health issue, whose incidence varies from 6.1% to 10.5% among critical patients^(1,5)^.

The development of PWs can be provoked by intrinsic and extrinsic factors. Intrinsic factors include age, nutritional deficiencies, tissue perfusion, urinary or fecal incontinence, loss of sensitivity, immunodeficiency, as well as chronic diseases (such as diabetes *mellitus* and cardiovascular diseases) and the use of certain medication. Extrinsic factors include pressure, shear, and humidity^(1,6)^.

The evolution of PWs is fast in most cases and is an indicator of low-quality assistance. Therefore, the nursing team must inspect the skin of the patients daily, evaluating their risk for developing these lesions and implementing preventive and/or healing interventions.

These workers are essential to prevent PWs and prevent their appearance, since they provide direct and constant care to critical patients. Consequently, nurses must recognize risk factors for PWs. After identifying these, they will be able to plan and implement activities targeted at minimizing their incidence and the complications that emerge from it, such as longer stays in the ICU and increased hospitalization costs.

Therefore, we believe that ascertaining the incidence and the factors of PW appearance in critical patients can contribute to improve the quality of nursing care, decreasing the hospitalization time of patients which would, as a result, reduce costs. Moreover, these data can aid in the implementation of evidence-based protocols to prevent PWs and educational programs to help reducing the incidence of this issue.

## OBJECTIVES

To identify the incidence of PWs in critical patients and its associated factors.

## METHODS

### Ethical aspects

The research followed all ethical guidelines prescribed by Resolution No. 466/2012 from the National Council of Health and was approved by the provider of the hospital setting of the investigation and by the Research Ethics Committee of the funding institution.

### Design, period, and place of study

This is an observational study, a retrospective cohort carried out in the Adult ICU of a medium-sized teaching hospital. The study was carried out by the STROBE guidelines (Strengthening the Reporting of Observational Studies in Epidemiology)^(7)^.

The ICU studied include six beds to receive patients in critical conditions due to clinical or surgical reasons. It is funded by the Single Health System, through complementary and/or particular systems. The team who works in the unit includes nurses, physicians, a physical therapist, nursing technicians, and general service personnel.

### Population; criteria of inclusion and exclusion

The study included the records of the 18-year-old or older patients admitted in the ICU from April 2018 to May 2019 with no pressure wounds. Patients whose records did not include complete data on the severity of their case were excluded, as well as those whose records contained no evaluation of the risk of PWs. The final convenience sample included 369 patients, who were monitored until the hospitalization reached its outcome or PWs appeared.

### Study protocol

Data were obtained from the analysis of the daily progress as described in the records. Information was recorded in an instrument elaborated by the researchers and divided in three parts: sociodemographic characteristics, clinical data, stratification of risk, and appearance of wounds.

The variables analyzed regarding sociodemographic data were: sex (male or female) and age (in years). General clinical data were: hospitalization time; clinical outcome (discharge, death, or transfer); medical diagnostic at time of hospitalization (grouped according with the titles of the International Classification of Diseases - ICD-10); score in the Simplified Acute Physiology Score III (SAPS III);; use of assistance devices such as nasoenteric catheter (NEC), triple lumen catheter (TLC), central venous catheter (CVC), vesical drainage catheter (CVD), peripheral venous catheter (PVC), invasive arterial pressure (IAP), tracheostomy (TQT), and orotracheal tube (OTT). Finally, the appearance of PWs during the hospitalization period and the risk for PW based on the daily score in the Braden Scale were found. The Braden Scale is formed by six components: sensory perception; humidity; mobility and activity; nutrition; friction; and shearing. Each component receives a score from 1 to 4. The total score is stratified in different levels of risk for the development of PWs. Patients with more than 16 points are considered to risk-free, in regard to developing PWs; from 12 to 16, the risk is moderate; and a result below 11 indicates a high risk^(8)^.

### Analysis of results and statistics

Data were input in the software Microsoft Excel 2007 and analyzed using the software SPSS 20.0. The incidence was calculated dividing the number of patients with PWs by the number of patients hospitalized in the unit during the study. Then, the normality of data distribution was verified using the Kolmogorov-Smirnov test, and descriptive statistics were calculated, including central tendency and variability measures, to characterize the patients who developed PWs and those who did not.

To evaluate the association of sociodemographic and clinical variables with the appearance of PWs in critical patients, a logistic regression was carried out. The presence of PWs was the dependent variable. The variables whose association with the PWs reached a level of significance of 20% (p<0.20) were included in the final logistic regression model. The Forward Method was used, and all variables in the final model showed statistical significance (p<0.05). This analysis allowed us to determine the effect regardless of association, using the odds ratio (OR), with a confidence interval of 95% and a significance level of 0.05. To verify the fitness of the final model, the Hosmer & Lemeshow, test was used considering that a good fit would be (χ^2^ = 3,99; p = 0,550).

The mean score of the Braden Scale of patients who did not develop PWs was compared to that of the patients who did using Student’s *t* for independent samples.

## RESULTS

From the 369 patients included in this study, 42 developed PWs, corresponding to an incidence of 11.4%. Most participants were male (202; 54.7%). The age of the patients varied from 21 to 100 years, with a mean of 72 years (standard deviation of 17.8). The main hospitalization causes were related to circulatory system diseases (137; 37.1%), followed by diseases in the respiratory (74; 20.1%) and digestive (46; 12.5% tracts). The hospitalization time in the sector varied from 1 to 64 days, with a median of 4 days (AIQ = 4 days). The SAPS III score median was 53.0 (AIQ 23.0; minimum of 25.0 and maximum of 105.0), and the estimated mortality median was 23.8% (AIQ 40.5%; minimum of 0.8% and maximum of 100.0%). However, the real percentage of patients whose outcome was death was 24.7% (91 patients).

The bivariate analysis showed association between PW and patients who were older, stayed longer in the ICU, and whose SAPS III was higher than the mean/median of the sample (p=0.035, p<0.001, p<0.001, respectively). Furthermore, PW was also associated with patients whose outcome was death (p = 0.019), according with Table 1.

**Table 1:** Bivariate analysis of the association between sociodemographic and clinic characteristics of critical patients and the appearance of pressure wounds, Viçosa, Minas Gerais, Brazil, 2019

Patient Characteristics	With PW (n = 42)		Without PW (n = 327)		OR	CI95%	*p* value
	n	%	n	%			
Sex							
Female	19	45.2	148	45.3	1		
Male	23	54.8	179	54.7	1.001	0.52-1.91	0.998
Age							
≤ 72 years	15	35.7	174	53.2	1		
> 72 years	27	64.2	153	46.7	2.047	1.05-3.99	0.035^*^
Time in the ICU							
≤ 4 days	08	19.0	209	63.9	1		
> 4 days	34	81.0	118	36.1	7.53	3.37-16.80	< 0.001^*^
SAPS III score							
≤ 53 points	06	14.2	179	54.7	1		
> 53 points	36	85.7	148	45.2	7.26	2.98-17.69	< 0.001^*^
Clinical outcome of the hospitalization							
Discharged from the ICU	21	50.0	210	64.2	1		
Death	17	40.4	74	22.6	2.30	1.15-4.59	0.019^*^
Transfer	04	09.5	43	13.1	0.93	0.30-2.85	0.899

PW – pressure wounds; OR – odds ratio; CI – confidence interval; SAPS III – Simplified Acute Physiology Score; ICU – intensive care units; *Statistically significant (p<0.05) according with Wald's test.

Table 2 shows that, regarding the health care devices used by the patients, the following showed statistically significant association with the development of PW: nasoenteric catheter (p<0.001), triple lumen catheter (p<0.001), central venous catheter (p=0.020), vesical drainage catheter (p<0.001), invasive monitoring of arterial pressure (p=0.023), tracheostomy (p<0.001), and orotracheal tube (p<0.001).

**Table 2:** Bivariate analysis of the association between health care devices used by the critical patients during hospitalization and the appearance of pressure wounds, Viçosa, Minas Gerais, Brazil, 2019

Variables	With PW (n = 42)		Without PW (n = 327)		OR	CI95%	*p* value
	n	%	N	%			
Nasoenteric catheter							
Absent	06	14.3	223	68.2	1	5.26-31.49	< 0.001^*^
Present	36	85.7	104	31.8	12.86		
Triple lumen catheter							
Absent	31	73.8	302	92.4	1	1.93-9.54	< 0.001^*^
Present	11	26.2	25	7.6	4.29		
Central venous catheter							
Absent	23	54.8	237	72.5	1	1.13-4.18	0.020^*^
Present	19	45.2	90	27.5	2.17		
Peripheral venous catheter							
Absent	05	11.9	33	10.1	1	0.30-2.26	0.716
Present	37	88.1	294	89.9	0.83		
Vesical drainage catheter							
Absent	03	7.1	158	48.3	1	3.68-40.12	< 0.001^*^
Present	39	92.9	169	51.7	12.15		
Invasive arterial pressure							
Absent	35	83.3	306	93.6	1	1.16-7.34	0.023^*^
Present	07	16.7	21	6.4	2.91		
Tracheostomy							
Absent	25	59.5	308	94.2	1	5.10-23.83	< 0.001^*^
Present	17	40.5	19	5.8	11.02		
Orotracheal tube							
Absent	12	28.6	233	71.2	1	3.04-12.62	< 0.001^*^
Present	30	71.4	94	28.8	6.20		

PW – pressure wounds; OR – odds ratio; CI – confidence interval; *Statistically significant (p<0.05) according with Wald's test.

Sociodemographic and clinical variables whose *p*<0.20 in the bivariate analysis went through a final logistic regression model. The patients who stayed more than four days in the ICU and used nasoenteric catheter, vesical drainage catheter, and had tracheostomy, had a higher chance of developing PW, as Table 3 indicates.

**Table 3:** Variables associated with pressure wounds in critical patients included in the final logistic regression model, Viçosa, Minas Gerais, Brazil, 2019

Variables	With PW (n = 42) n	Without PW (n = 327) %	OR	CI95%	*p* value
Time in the ICU (n)					
≤ 4 days	08	209	1	1.15-7.78	0.025^*^
> 4 days	34	118	2.99		
Nasoenteric catheter (n)					
Absent	06	223	1	1.40-10.38	0.009^*^
Present	36	104	3.81		
Vesical drainage catheter (n)					
Absent	03	158	1	1.31-17.38	0.018^*^
Present	39	169	4.78		
Tracheostomy (n)					
Absent	25	308	1	1.48-8.97	0.005^*^
Present	17	19	3.64		

PW – pressure wounds; OR – odds ratio; CI – confidence interval; *Statistically significant (p<0.05) according with Wald's test.

There was a statistical difference in Braden scale results (p<0.001) between the mean score of patients who developed PW (14.2) and the mean score of those who did not (12.3).

In regard to the stratification of PW risk, patients classified as low risk had a lower chance of developing these wounds (OR 0.22; CI95% 0.06-0.72). Patients classified with a high risk of developing PW were three times more likely to have the issue, as Table 4 shows (OR 3.22; CI95% 1.66-6.23),

**Table 4:** Bivariate analysis of pressure wounds risks of critical patients with and without pressure lesions, Viçosa, Minas Gerais, Brazil, 2019 (n = 369)

Braden scale	With PW (n = 42)		Without PW (n = 327)		OR	CI95%	*p* value
	n	%	N	%			
Low risk (more than 16 points)	03	7.1	86	26.3	0.22	0.06-0.72	0.012*
Moderate risk (12 to 16 points)	19	45.2	169	51.7	0.72	0.40-1.47	0.433
High risk (11 or less points)	20	47.6	72	22.0	3.22	1.66-6.23	0.001*

PW – pressure wounds; OR – odds ratio; CI – confidence interval; *Statistically significant (p<0.05) according with Wald's test.

## DISCUSSION

In this study, the incidence of PW in critical patients was 11.4%, considered low when compared with literature, according to which common values are 19.5% and 29.5%^(9-10)^. This result is a consequence due to the good practices adopted by the nursing team in the ICU studied, which contributed for the quality of the nursing care. These practices are: decubitus changes, the use of materials and equipment that redistribute the pressure (cushions, mattresses, calcaneus protection), maintenance dry and with hydrated skin, in addition to the daily evaluation of the risk of developing PW of all patients through the use of the Braden Scale^(11)^.

The management of PW risk is frequently carried out using scales that track more vulnerable patients and aid in the identification of risk and in the making of decisions of nurses. Identifying the PW risk using formalized evaluations is an important stage of any protocol to prevent this problem, and its formalization is considered to be an important stage of any protocol to prevent this problem. It is recommended in clinical practice directives^(12)^. The use of scales to measure the risk of PW by the nursing team is a relevant action of care and an effective mechanism to reduce the incidence of this event^(8,13)^. However, identifying and classifying the risk should not be the only action in this regard. Actions should be individualized and targeted at each patient, according with the identification of risk factors and of the implementation of measures to that minimize their occurrence^(8)^.

Regarding risk factors, the time of permanence of patients in the ICU was higher than the median of the sample (four days) and associated with the development of PW. A study carried out in the ICU of a teaching hospital in the northeast of Brazil found that patients hospitalized for long periods are approximately four times more likely to develop PW (OR=3.92). Furthermore, it is well known that the time in the ICU is directly related with the severity of the case of the patients and their need for health care^(14)^.

In this study, in addition with the time in the ICU, the use of assistance devices such as VDC, NEC, and tracheostomy also had statistical associations with PW. The same result was found in other studies^(15-16)^. It stands out that, in addition to the devices identified in this research, other devices are also risk factors for the development of PW according with other studies, such as the use of splints, orthopedic devices^(17)^, endotracheal tubes^(17-18)^, catheters for oxygen administration^(17-18)^, and compression stockings^(19)^. These devices can cause heat, humidity, and pressure on the skin of patients, predisposing them to develop PW. Although these are heterogeneous materials, which are useful for many ends and located in different parts of the body, the similarity is in the fact that all are placed on soft tissues and can cause PW or friction. In addition, it is worth highlighting that the use of invasive devices restricts the mobility of patients, keeps them restricted to the beds for longer, and, as a consequence, increases the risk of PW^(20)^. Specifically in regard to the association with the use of NEC, studies indicate the existence of a relation between the nutrition of patients and skin integrity, PW, and wound healing, highlighting the relevance of adequate nutrition, with the adequate amount of calories and proteins^(21-22)^.

Scientific literature shows that the incidence and prevalence of PW related with the use of health care devices is 12% (CI95%: 8-18) and 10% (CI95%: 6-16), respectively^(23)^. These findings reiterate that this is a significant public health problem, especially as these lesions can compromise the wellbeing of the patients and increase the cost of care. In this context, it is essential to implement a plan of care that prevents the occurrence of PW and promotes the best attention possible for the needs of the patient.^(8)^.

The use of the Braden Scale by nurses in daily patient care is an important tool to evaluate and implement preventive measures regarding PW^(8,13)^. Regarding the category “Braden Scale category”, patients with a high risk for PW in this study were three times more likely to develop it. Therefore, it is paramount to implement preventive actions that contribute with the reduction of PW and secondary damage. Therefore, the multiprofessional team provide integral care and use the risk prediction scale, identifying the patients under risk of developing wounds as early as possible and tracing an effective health care plan, aiming at minimizing the occurrence of PW in critical patients.

It is noteworthy the importance of systematized and individualized care, directed at the identification of the PW risk and at the implementation of preventive measures. A study with patients hospitalized in an ICU in the Brazilian northeast showed that the use of PW scales validated in accordance with the nursing diagnosis (ND) of “Risk of pressure wound” improves nurse’s critical judgment in regard to motives that increase the risk of PW. Furthermore, they allow the nurses the understand better the aspects that can be modified based on the identification of risk factors, including the population at risk and their conditions^(8)^.

Different studies address several potential conducts to prevent PW. Among them, the actions of the nurse in the development of individualized care that continuously evaluates the skin of the patient until the outcome of hospitalization^(8,24)^. In addition to constant nurse evaluation, preventive interventions include: moving the patient in bed and protecting bone prominences, to reduce vascular involvement provoked by the pressure on the bed; and keeping a clean, dry, hydrated skin using essential fatty acid creams that can be a barrier against humidity^(24)^.

Despite the evidences described, it is still challenging for ICU nurses to systematize care to prevent PW and treat cases. For the assistance to be successful in doing so, it is important to acquire scientific knowledge and increase our understanding of the care associated with good health practices, especially in the elaboration and execution of preventive interventions^(25)^. Furthermore, it is essential for nurses to identify the real needs of the patients, including a broad evaluation of biopsychosocial and spiritual aspects coupled with the practical experience of scientific knowledge, so more assertive nursing diagnoses can be reached. As a result, it would be possible to plan actions to reduce PW incidence and reach the nursing outcomes expected^(24)^.

### Study limitations

Limitations of this study include the fact that the research was carried out in a single general adult ICU and the fact that data collection was carried out from medical records. Also, since the patients were not directly evaluated, it was impossible to evaluate the main anatomic regions affected by PWs and the stage of the wounds, since the records evaluated did not include this information.

### Contributions to the Field of Nursing

We believe this study can contribute for the practice of nursing as it uncovers the incidence of pressure lesions and its associated factors by analyzing a representative sample of critical patients, whose care is mostly a responsibility of the nursing team. Considering that pressure wounds are indicators of low-quality nursing assistance, it is essential for the nurse to recognize the factors associated with their occurrence and to implement interventions to minimize their incidence and the consequent complications it provokes, such as longer permanence in the sector and increased hospitalization costs. Moreover, we expect the dissemination of the findings of this study to improve nursing diagnostic reasoning in this regard, leading to better results through the implementation of assertive interventions.

## CONCLUSIONS

The incidence of PW among critical patients in this study was 11.4%. It was associated with more than four days permanence in the ICU, tracheostomy, and the use of nasoenteric catheter, and vesical drainage catheter.

Although the incidence of pressure wounds was low, it is essential to consider studying the factors associated with the appearance of PWs in critical patients, especially in regard to the use of health care devices, since national literature is lacking in regard to this subject. Not only this is important to decrease the incidence of this nursing issue and to help nurses to implement preventive strategies based on adequate evidence, but it is also relevant to develop and test risk-evaluation models in this population.
